# A personalized care plan in chronic care: implementation and evaluation

**Published:** 2012-09-04

**Authors:** Jeanny Engels, Marjolein Rebel, Doortje Boshuizen

**Affiliations:** Vilans, Catharijnesingel 47 Postbus 8228, 3503 RE Utrecht, The Netherlands; Vilans, Catharijnesingel 47 Postbus 8228, 3503 RE Utrecht, The Netherlands; Vilans, Catharijnesingel 47 Postbus 8228, 3503 RE Utrecht, The Netherlands

**Keywords:** personalized care plan, self-management, vascular risk, multidisciplinary team, chronic care

## Abstract

**Purpose:**

Implementation and evaluation of a personalized care plan for approximately 350 people with (an increased risk of) cardiovascular disease in ten general practices in the Netherlands.

**Context:**

The ‘Healthy Vessels’ (‘Vitale Vaten’) care standard of 2009 describes the optimum care for people with (an increased risk of) cardiovascular disease and is based on the Chronic Care Model. New: working with a personalized care plan, with detailed attention for the promotion of self-management and shared decision-making (SDM). This requires patients to adopt a more active attitude, with a more coaching role from care providers. Vilans has developed the personalized care plan for cardiovascular disease (the booklet ‘Zorgplan Vitale Vaten’) and the personalized care plan for diabetes and for COPD in 2011. In 2011 Vilans also started with the development of a general care plan for patients with multi morbidity diseases.

**Data sources:**

Patients: quantitative survey with a written questionnaire sent to approximately 75 patients. Baseline and end points for 40 patients, plus in-depth interviews with eight patients.

**Care providers:**

Quantitative survey with a written questionnaire sent to 45 care providers. Baseline and end points for 22 care providers, plus in-depth interviews with 10 care providers.

**Case description:**

The personalized care plan is produced by a shared decision-making process and consists of:

- A prioritised list of the patient’s SMART objectives
- A personalized plan for achieving those objectives
- Agreements concerning what the patient will do himself/herself and the support or advice needed
- Agreements concerning contact to review the progress (how and when)

The patient or the care provider notes the plan in the patient’s booklet (the ‘Zorgplan Vitale Vaten’=‘Healthy Vessels Care Plan’). This booklet also contains information about the risk factors for cardiovascular disease, the importance of the patient adopting an active role, measurement values, medication and the patient’s care providers.

Advisers from Vilans, the knowledge centre for long-term care in the Netherlands, provide participating organisations guidance for the implementation of the personalized care plan with: work conferences, supporting products and monthly support phone-calls or e-mails.

The project consists of the following phases:

- Jan 2010 to Jun 2010: development of materials
- Jun 2010 to Oct 2011: implementation and evaluation in ten general practices
- Nov 2011 to Feb 2012: project completion and reporting
- The results will be available in February 2012

**The study questions in this project are:**

1. What effects does the personalized care plan have on the level of self-management of the patients?
2. What effects does the personalized care plan have on professionals in a multidisciplinary team?
3. Do the effects also apply to ethnic minority patients and patients with a low socio-economic status?

**(Preliminary) conclusions:**

- Self-management/Shared Decision Making is difficult to implement. Regular feedback and joint learning are needed.
- It is helpful when agreements between the patient and the care provider are made concrete: writing things down makes a difference.
- Variable response from patients: ranging from ‘good to know you have something to fall back on’ to ‘the idea of writing down personal objectives makes me feel a bit nervous’.
- The personalized care plan does not seem suitable for all, in particular not for the elderly, for those of low socio-economic status, and for ethnic minorities.

**Discussion:**

Health care professionals are used to take care of patients with chronic diseases. They are very willing to help and give patients some advice about how they can prevent a chronic disease or have a good life with a chronic disease. During the conferences and phone calls we have with them, we see that the focus is more on caring instead of sharing and self-management. It frustrates professionals when patients do not behave the way they tell them to. They do not know how to handle or turn the conversation into self-management and rather fall back in their roll of caring. It seems necessary to get feedback on a regular basis so they can explore new ways of self-management support together in a multidisciplinary way.

Self-management support is more successful when professionals are working together, looking for ways to take into account the perspective and expectations of the patient as well as those of the professional. The personalized care plan can help patients and professionals exploring their new roles.

There are some relevant questions concerning personalized care plans in practice which we cannot yet answer. We would like to discuss these essential questions with the participants of INIC12. For example:

- How important is it for patients to have a personalized care plan? Does it support them in making decisions concerning their health in daily life? In what way can a digital care plan provide help?
- Do professionals improve their caring and communication with patients with chronic diseases when they use a personalized care plan?
- Is it more successful when one professional is the central care provider for a patient?
- What are good ways for integrating personalized care plans in usual care? Does it take more time in comparison to regular care?
- How to create possibilities for professionals so they can regard the personalized care plan as an important topic in chronic care? We see it is difficult for a small group of patients. How to implement the personalized care plan for all the patients with a chronic disease?
- What do the answers to these questions mean and does individual care planning change the health care process in such a way that self-management can flourish?

Powerpoint presentation available at http://www.integratedcare.org at congresses – San Marino – programme.

